# Postural instability after lumbar spinal surgery: are there any predictive factors? A case control study

**DOI:** 10.1186/s41016-018-0147-2

**Published:** 2018-12-11

**Authors:** Domenico Chirchiglia, Pasquale Chirchiglia, Domenico Murrone

**Affiliations:** 1 Department of Neurosurgery, University of Catanzaro, Campus Germaneto, VLE Europa, 88100 Catanzaro, Italy; 2 Department of Neurosurgery, Divenere Hospital, 70121 Bari, Italy

**Keywords:** Spine disease, Lumbar pain, Postural instability

## Abstract

**Background:**

The surgical spinal degenerative pathology mainly concerns the herniated intervertebral disks. Surgery is indicated when the pain becomes chronic and intense, and when motor signs appear. The results are positive in about 90% of cases, leading to the solution of the problem. However, an estimated percentage of 4% to 20% reported residual pain and postural instability after the surgical treatment of discectomy.

**Method:**

We have examined a sample of patients, retrospectively registered, undergoing surgical treatment for degenerative lumbar disease. Some of them developed postural instability. They were subjected to cycles of postural gymnastics. Postural gymnastics has proved to be a tool capable of solving unstable post-surgical posture. It included an exercise of breathing, one or two of muscular distension, one of muscular reinforcement, and one of postural correction. We used an evaluation form we created in agreement with the physiatrist for postural exercises that was based on some basic parameters such as muscle and respiratory function. At each cycle, a score was attributed to the performance of muscular and respiratory exercise to evaluate the function and therefore the degree of instability (1–3 = mild, 4–7 = medium, 8–10 = severe).

**Results:**

Results were satisfactory, with return to normal posture. The improvement of postural instability has been demonstrated both by the score of the evaluation forms that have highlighted the transition from a state of severe intensity to one of normality and by a clinical aspect, concerning the static and dynamic posture.

**Conclusions:**

The postural instability has a multifactorial genesis, and different mechanisms are involved: the vertebral bone structures and the pelvis, the paraspinal muscular structures, and the nerve structures. These structures are altered after surgery due to predisposing factors, and for the action of conditions acquired as obesity.

## Background

The degenerative lumbar spinal pathology is very frequent, especially the disc herniation. This disorder is a cause of pain, worsening of the quality of life and loss of working days. Surgical treatment is indicated when the pain becomes chronic, and if motor symptoms appear.

Results are satisfactory in 70% to 95% of cases, especially for the disappearance of lumbar pain [1, 2]. After surgery, there is an aspect not sufficiently evaluated: the post-surgical posture stability. It is known that a static or dynamic posture is ensured by various functions, such as nervous, muscular, and bone. A posture is stable when there is integrity of the structures of the spine, especially the disc and the nerve root. Under normal conditions, the intervertebral disc (which is a buffer between the vertebrae) makes harmonious the movements of the lumbar spine. The spinal nerve is the starting point of the movement, with the help of muscle tone. In the case of disc diseases, such as hernias, it can happen that the equilibrium between vertebral structures is altered, causing an alteration of the posture.

The purpose of this study is to determine why, after lumbar spinal surgery, postural instability (PI) arises. This is a useful message, because instability can be cured, successfully, through a simple treatment of postural gymnastics.

The literature offers some data: PI, after spinal surgery, has an incidence between 4 and 20% [3]. Various factors affect postural stability, such as immediate return to physical activity, the presence of pre-existing vertebral alterations, excessive weight, and old age. These should be considered predictive factors for the onset of post-surgical PI.

## Methods

We have studied retrospectively 50 cases of patients suffering from low back pain or sciatica. All of them had been operated for lumbar herniation disc at University Hospital of Catanzaro, Italy. The sample comprised 32 females and 18 males, aged between 30 and 58 years. The most affected level was L4-L5 (40 patients). Patients were recommended to have a rest period of at least 2 weeks after surgery. A follow-up of 5 years showed the success of the surgery in 47 patients, in which there was complete disappearance of the pain, while three patients (L4-L5 level in two patients, L5-S1 in one patient) reported residual lumbar pain of mild intensity in 2 years of control. They had more than 50 years of age. Lumbar spine MRI with gadolinium showed in a patient scars at the level of the operative outbreak. The pain was associated with unstable posture, condition of improper position of the body, both from firm and during movement, with tendency to loss of balance and fall. Postural instability appeared getting out of bed and starting walk. Walking, the patients manifested muscular weakness accentuated by the fatigue. Radiographic examinations of the lumbar spine, in flexo-extension, showed no alterations of the morphology of the vertebral bodies and their alignment. Radiography of the pelvis and hip bones showed no signs of pathology. Laboratory tests were performed, including muscle enzymes, creatine-kinases, and creatine-phospho-kinases, which were normal. Neurological examination showed normal tendon reflexes, as well as trophism and muscular tone. It should be noted that these three patients had a weight exceeding the norm (about 75–78 kg, in an average height of 160 cm) (Table 1).

**Table 1:** Parameters for postural instability

Patient	Sex	Year	Weight/height (kg/cm)	Disease	Location	Time postural exercise/month	Criteria of postural instability: clinical motor signs, both static and dynamic
1	F	55	90/155	Disc herniation L4/L5 left	Lumbago	One	Clinical motor signs present. Evaluation form score = 8
2	M	65	100/165	Disc herniation L4/L5 left	Left Lumbo sciatica	One	Clinical motor signs present. Evaluation form score = 8
3	M	67	97/170	Disc herniation L5/S1 right	Right Lumbo sciatica	One	Clinical motor signs present. Evaluation form score = 8

We used an evaluation form we created in agreement with the physiatrist for postural exercises that was based on some basic parameters such as muscle and respiratory function. At each cycle, a score was attributed to the performance of muscular and respiratory exercise to evaluate the function and therefore the degree of instability (1–3 = mild, 4–7 = medium, 8–10 = severe).

So, conservative-rehabilitative treatment was recommended, through repeated cycles of physiotherapy, including stretching and muscular reinforcement. Lumbar postural exercises were initiated. They, divided into four series of difficulties gradually increasing, were carried out under the guidance of a physiotherapist, for an average period of 4 weeks. After this period, the patient had acquired the correct execution procedures and therefore continued to carry out the work program at his domicile. Each series included an exercise of breathing, one or two of muscular distension, one of muscular reinforcement, and one of postural correction. The items were as follows: first week, (A) thoracic respiration in supine position; (B) distension of the hip flexor muscles in supine position; (C) reinforcement of the abdominal muscles; and (D) postural correction in supine position. Second week: (A) abdominal respiration in supine position; (B) Ischio-peroneal-tibial muscles distension in supine position; (C) reinforcement of the abdominal muscles; and (D) postural correction in a seated position. Third week: (A) abdominal and thoracic respiration combined in supine position; (B) distension of the gastrocnemi muscles in the proximal and distal insertion; (C) abdominal reinforcement in supine position; and (D) posture correction in upright position. Fourth week: (A) respiration combined in supine position transferring air from chest to abdomen; (B) lumbar masses distension in a seated position (a flexed hip and the other extended); (C) abdominal reinforcement in axial position with flexed legs; (D) buttocks reinforcement; and (E) posture correction in upright position. The patients underwent electromyographic examination, showing signs of radiculopathy, which was moderate in each of them.

## Results

The improvement of postural instability has been demonstrated both by the score of the evaluation forms that have highlighted the transition from a state of severe intensity to one of normality, concerning the static and dynamic posture.

After two cycles of 8 weeks therapy, postural instability improved considerably, and currently, patients report an improvement in the transition from the supine to the upright position from and during movements, showing a more stable gait.

## Discussion

Lumbar spinal instability is known to be associated with chronic low back pain as one of its important causes. The incidence of spinal instability is difficult to determine partly because the lack of a universally accepted definition.

The percentage of patients with low back pain arising because of spinal instability ranges from 13 to 30%. Other authors report a range from 4 to 20% [3, 4]. Today, it is a less frequent condition, after surgery, thanks also to the most sophisticated surgical and neuroimaging techniques.

Spinal instability is defined as the inability of the spine to maintain its position under physiological loads. It seems that spinopelvic alignment is of great value for maintaining a good posture and subsequently preventing low back pain [5].

Lumbar discectomy is the most common spinal surgery treatment type. Usually, patients were recommended to reduce post-surgical activity to reduce the risk of disc re-herniation and progressive instability. Such a practice delayed the return to work. On the other hand, patients were advised do not reduce post-surgical activity as deemed unnecessary.

With the increase in the average age of life, in particular people over 65 years, the incidence of degenerative lumbar spine stenosis grows proportionally. Various parameters of the spinopelvic structures are used to predict surgical treatment outcomes in patients with degenerative spine diseases. There are no unified protocols for evaluation of surgical treatment outcomes in elderly patients.

Traditional procedures concerning post-surgical activity limitations, following lumbar spine surgery, may reduce the risk of progressive instability or lumbar disc re-herniation [6]. Prolonged sitting has been suggested to decrease lumbar lordosis, increase spinal loading and muscle activity, and contribute to accelerate disc degeneration and low back pain. Bono et al. [7] did not found differences in outcome measures between patients who observed 2 weeks or 6 weeks of activity reduction, even though finding differences in re-herniation rates and instability was underpowered. First studies investigating the results after the removal of post-surgical restrictions reported no increased risk of re-herniation or re-operation in patients not observing activity limitations following lumbar discectomy surgery, but these studies lacked comparison groups and therefore did not have a true scientific value [8, 9]. Currently, lumbar discectomy is minimally invasive, resulting in less tissue destruction and decreasing the importance of limiting activity. In a recent study, patients were recommended no sitting restrictions (22%) or sitting as comfort (40%) [10]. The majority (84%) was recommended restricted raising.

In another study, patients did not observe post-surgical activity restrictions returning to work earlier. The mean time to return to work was 1.2 weeks. Currently, recommendations suggest four to 16 weeks of absence of work following lumbar discectomy surgery [11].

Low back pain is considered one of the main causes of disability in the world [12]. Intervertebral disc degeneration is a frequent cause of low back pain. Lumbar discectomy for symptomatic intervertebral disc herniation is the most common spine surgical procedure. Activity restrictions have traditionally been recommended after the surgical treatment and patients delayed returning to work for 4 or more weeks [13]. A better knowledge of the role of post-surgical restrictions will allow a uniformity of post-surgical treatment and will allow patients to return to work more rapidly, thus reducing the social and economic burden of this condition. But are there predictive factors of unstable posture after surgery? Vertebral bone content and its geometry, as intervertebral disc width, frontal area and facet joint tropism, were found to be important predictors of postural instability, following laminectomy, suggesting that these variables could predict the possible development of post-surgical instability. Prediction of residual strength and stiffness of a spinal segment after laminectomy is useful for a surgeon to decide whether or not to use instrumented fusion techniques [14]. Biomechanical effects after laminectomy can be worded by the torsion load of the spine. Torsion loads may cause and progress disc degeneration and may even cause failure of a segment [1, 7, 8, 10]. It has been shown in vitro experiments that laminectomy results in a substantial decrease of torsion stiffness and torsion moment to failure of lumbar spinal segments [14]. For shave loads, the biomechanical behavior of a spinal segment following laminectomy has been shown to depend on disc degeneration, facet joint degeneration, Modic changes, Schmorl’s nodes, intervertebral disc and pedicle geometry, and facet joint angles [5, 15]. This may also hold for torsion strength and stiffness following laminectomy. If true, such variables may aid surgical decision-making on the need for instrumented stabilization of the spine during surgery. Spinal instability is a term coined to describe abnormal movement between one vertebra and another. When a disc degenerates, it loses tension, which allows the disc to bulge and permits increasing movements to take place between the vertebrae [16]. The loss of disc height causes the facet joints to displace and replace beyond their correct congruent alignment. The replacing and abnormal slipping of the facet joints induces arthritic overgrowth of the joints and the production of bone spurs around the joint margins. Abnormal sliding between vertebrae may occur during flexion, lifting, or extension, causing significant back pain, described as a stabbing pain. This usually occurs when the sufferer is getting up from the chair and standing upright [17, 18].

Considerations on the three cases we observed, due to post-surgical PI:

The number of patients who developed post-surgical postural instability following laminectomy with discectomy, for disc herniation, is small: three patients out of 50.

Degenerative, pre-surgical vertebral abnormalities concerning the vertebral bodies such as Schmorl and Modic were absent. We remember that both intra-spongious nodules of Schmorl and Modic lesions are degenerative osteochondrosical processes, which alter the normal structure of the vertebral bodies. They contribute to the creation of spinal discal diseases. The Modic classification is based on MRI imaging of the degenerative subchondral process, in the various stages of modification of the intraspongious signal: fibrovascular degeneration with edema signal (Modic 1), fat substitution (Modic 2), and sclerotic degeneration (Modic 3).

To note, no anomaly of the pelvic bones was detected. There was a formation of adherent cicatricial tissue, at the level of the intervertebral space operated. The patients were subjected to a 4-week postural gymnastics cycle, after which their instability greatly improved. At present, the three patients have resumed their normal stability. So, what caused this complication?

We can suppose that there is an individual predisposition to produce post-surgical alterations, for a probable tissue vulnerability: the scar formed in the operative focus is suggestive.

The excess weight has its negative role, weighing on a spine, made already unstable for the surgery.

The importance of physiotherapy which is decisive in treating symptomatology and restoring normal posture.

## Conclusions

Postural instability that occurs after surgery of lumbar disc disease is not a frequent event. It limits the movements, both at rest and in walking, associating with mild lumbar pain. In the cases observed, we can hypothesize that there could be an individual predisposition to create such a complication, with the tendency to create a post-surgical scar, probably due to a tissue factor. The excessive weight worsens the condition.

The positive note is the effectiveness of postural gymnastics, which in our cases has immediately proved to be effective. Ultimately, this study has an importance for preventive purposes. The knowledge of predictive factors, in particular the clinical ones, such as obesity, old age, but also radiographic ones, must suggest to the surgeon the option to choose. He will have to put into account, in the case of surgical treatment, the possibility that one can develop a postural instability, although reversible.

## Abbreviations

MRI: Magnetic resonance imaging
PI: Postural instability
